# Central serous retinopathy associated with topical oral corticosteroid use: a case report

**DOI:** 10.1186/s13256-019-2143-3

**Published:** 2019-07-02

**Authors:** Preston O’Brien, Ryan C. Young, Shelley Day Ghafoori, C. Armitage Harper, Robert W. Wong

**Affiliations:** 1 Austin Retina Associates, 801 W. 38th St., Suite 200, Austin, TX 78705 USA; 2 Department of Surgery and Perioperative Care, Dell Medical School, University of Texas, Austin, USA

**Keywords:** Pigment epithelial detachment, Central serous chorioretinopathy, Fluocinonide, Topical oral gel

## Abstract

**Background:**

Oral topical corticosteroid gels are widely used in dental medicine. Case studies of central serous retinopathy have been reported following administration of corticosteroids, but none so far coinciding with the use of topical fluocinonide gel. This case report further contributes to the database of potential risks of corticosteroid use.

**Case presentation:**

A 40-year-old South Asian woman presented with decreased vision, pigment epithelial detachments, and serous retinal detachments in both eyes 1 month after starting treatment with topical fluocinonide 0.05%, a topical oral corticosteroid gel. Her condition resolved 6 months after discontinuing the use of the steroid.

**Conclusions:**

To the best of our knowledge, this is the first case of idiopathic central serous retinopathy associated with the use of oral fluocinonide gel. Discontinuing the use of the steroid may result in resolution of the serous retinal detachment and improvement of visual symptoms. Patients and their doctors who prescribe this medication should be aware of this association.

## Background

Patients with idiopathic central serous retinopathy (CSR) develop a serous detachment of the sensory retina in the macula caused by a leakage of serum into the subretinal space [1]. CSR has a predilection for men aged from 25 to 50 years, but may occur in women or individuals at an older age. Although the pathogenesis remains unknown, risk factors associated with CSR include type A personality [2], Cushing disease [3, 4], pregnancy [5], and exposure to exogenous corticosteroid use [6]. Previous cases of CSR have been reported following the use of systemic [7], epidural [8], intranasal [9], peri-ocular [10], intra-articular [11], and topical dermal corticosteroids [12–15], along with one case of 0.1% oral topical triamcinolone acetonide gel [16].

To the best of our knowledge, we report the first case of CSR associated with the use of a topical oral fluocinonide gel for the treatment of post-surgical oral-mucosal inflammation following dental surgery.

## Case presentation

**Fig. 1:**
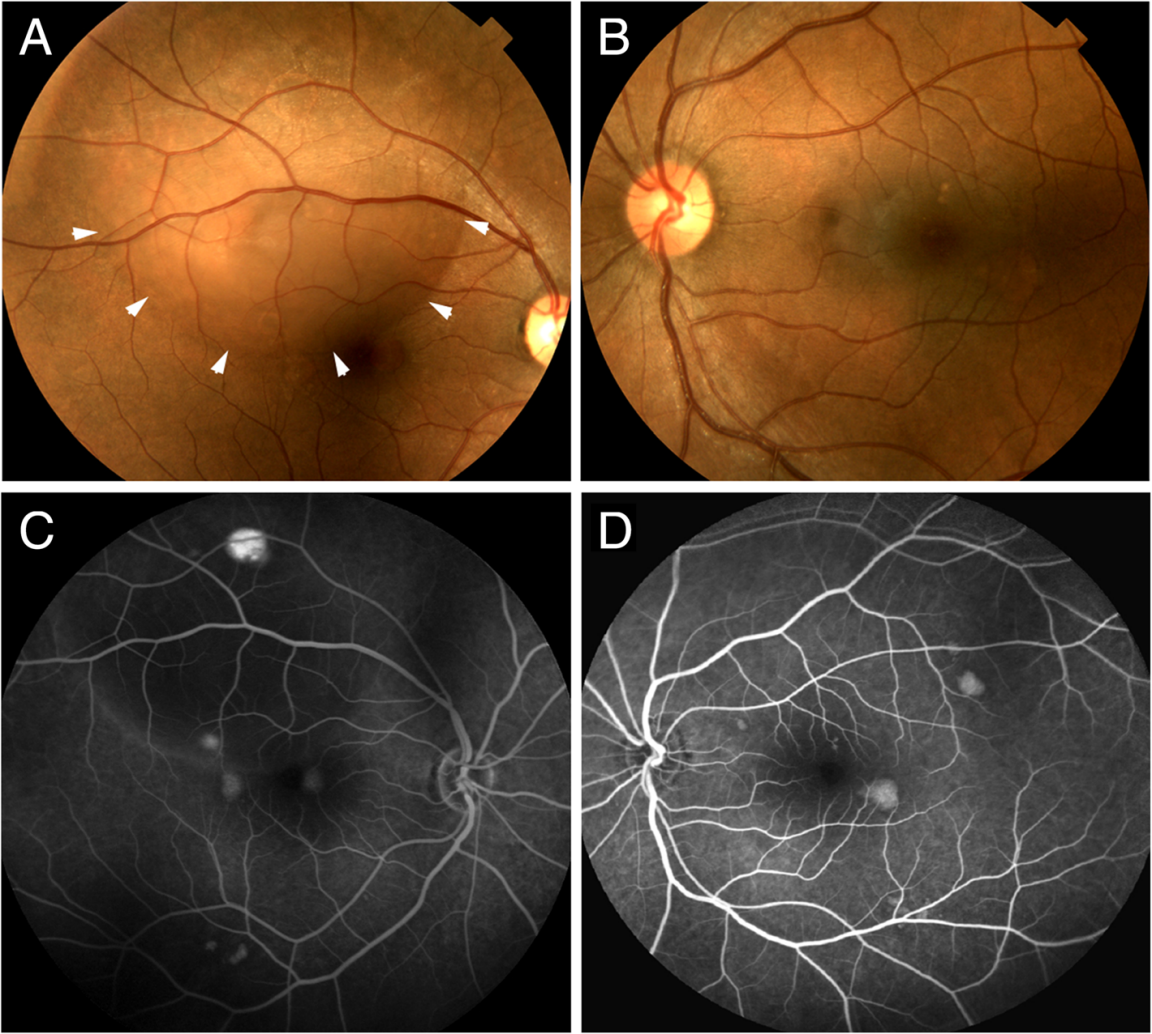
**a** Fundus photograph of the right superior macula showing subretinal fluid (*white arrowheads*), pigment epithelial detachments, and subretinal precipitates. **b** Fundus photograph of the left macula showing several pigment epithelial detachments. **c** Late phase fluorescein angiogram of the right eye showing pooling of dye within the pigment epithelial detachments and early filling within the subretinal fluid. **d** Late phase angiogram of the left macula showing pooling of dye within the multiple pigment epithelial detachments

A 40-year-old South Asian woman presented with blurring of vision, cloudiness, and a dark spot on her right eye for 1 week. She stated that she had been using a topical oral gel medication, fluocinonide 0.05% oral gel, twice a day for the past month as prescribed by her dentist for mucosal inflammation following oral surgery. She denied specific stressors in her life, current pregnancy, or other exogenous steroid medication use. She did not present with a history of hypertension; other past medical, surgical, family, and social histories were reviewed and were noncontributory. Her best corrected visual acuity measured 20/25–2 in her right eye and 20/20–2 in her left eye. The intraocular pressures were normal and anterior segment examinations in each eye were unremarkable. Posterior segment examination of her right eye (Fig. 1a) showed a large serous retinal detachment in the superotemporal macula with multiple associated pigment epithelial detachments located inferonasal to the fovea and another in the inferotemporal macula. The posterior segment of her left eye (Fig. 1b) revealed multiple pigment epithelial detachments within the macula. There was no evidence of intraocular inflammation in either eye.

**Fig. 2:**
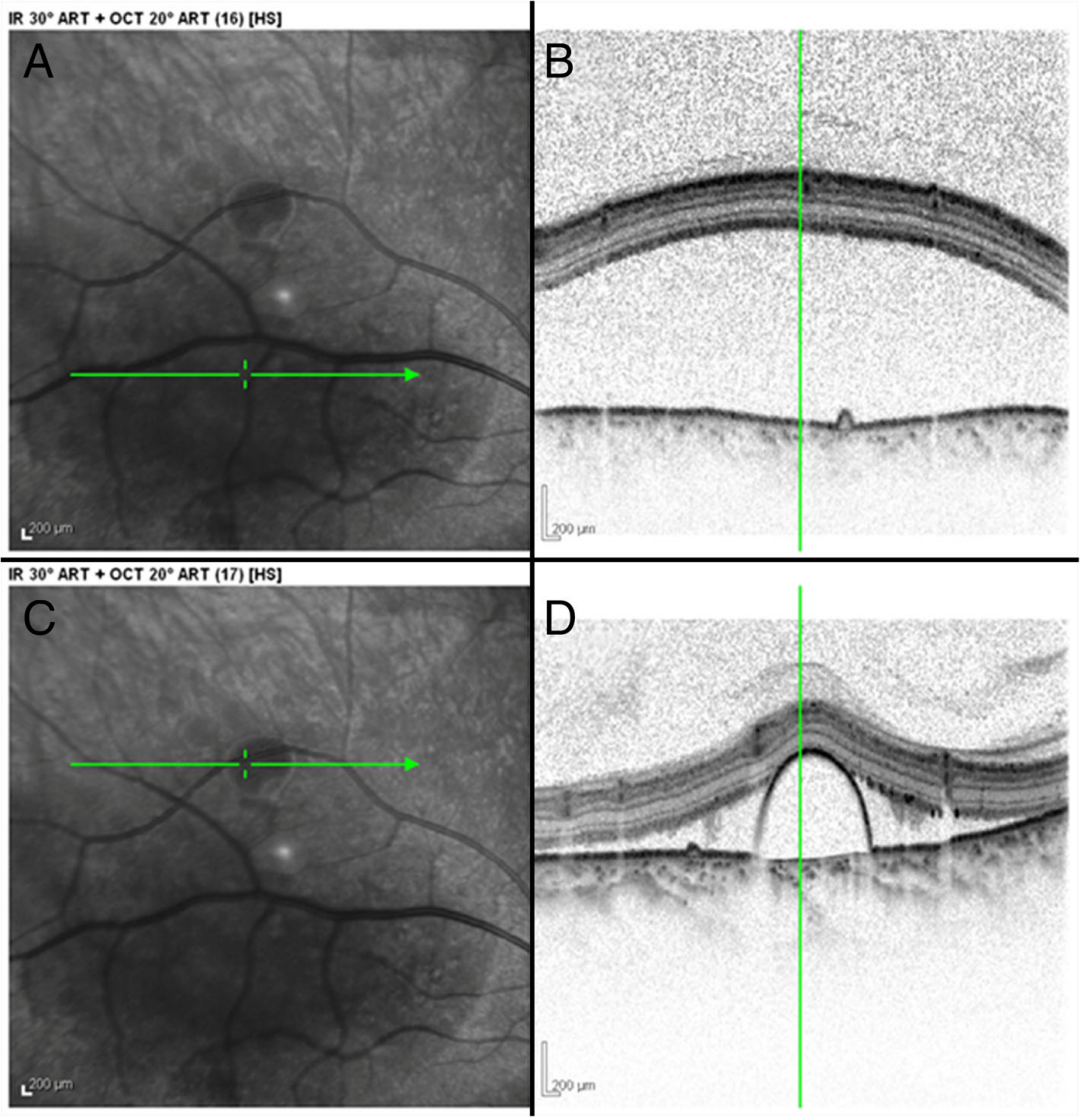
High resolution spectral domain optical coherence tomography images of the superior right macula at time of presentation. **a** En face infrared image of superior macula where the *green line* relates to the image seen in (**b**). **b** Cross-section optical coherence tomography image showing subretinal fluid in superior macula. **c** En face infrared image of superior macula where the *green line* relates to the image seen in (**d**). **d** Cross-section optical coherence tomography image showing subretinal fluid surrounding a serous pigment epithelial detachment

**Fig. 3:**
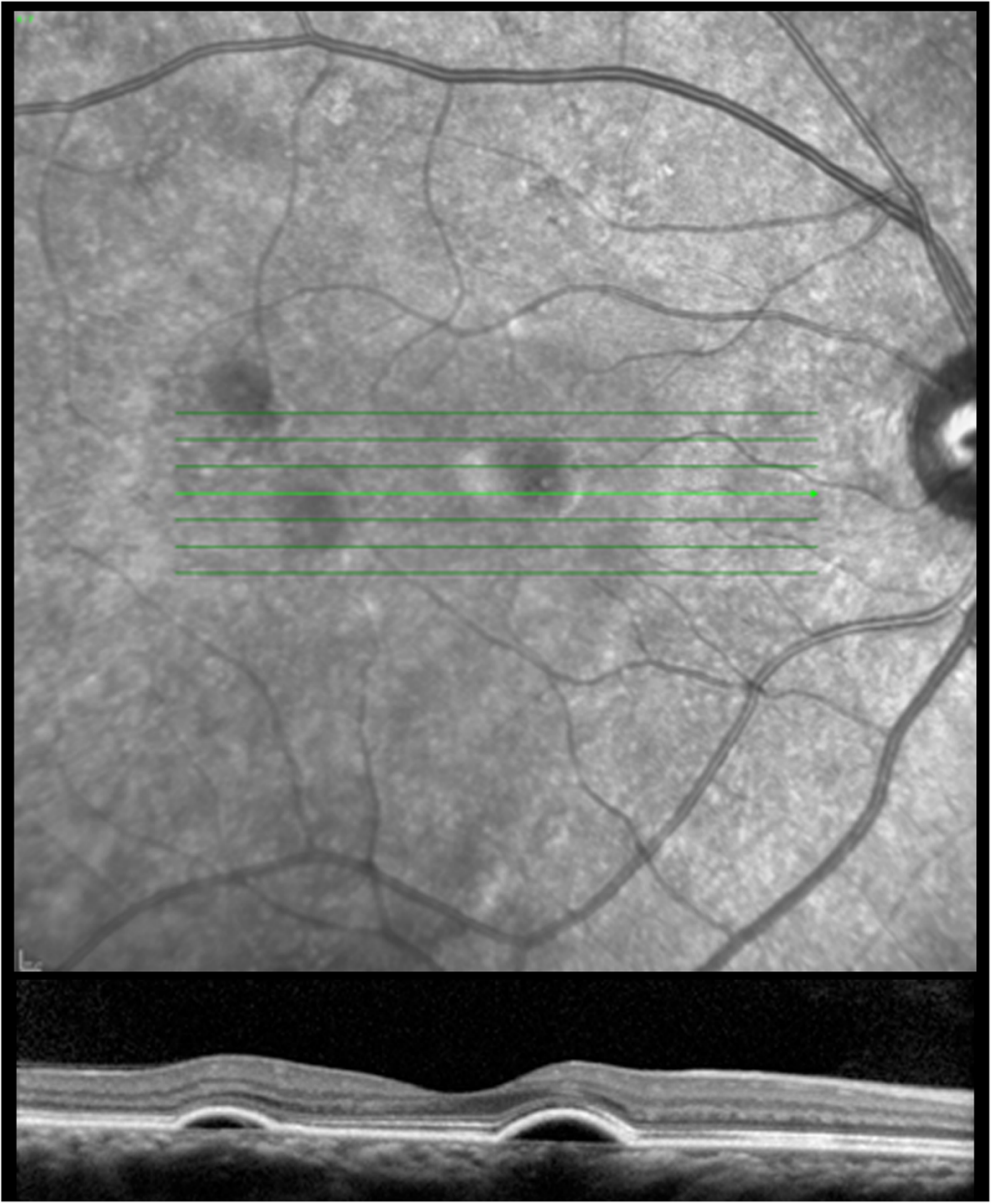
High resolution spectral domain optical coherence tomography images of the right macula upon 2-month follow-up examination. *Upper panel* details en face infrared image showing multiple pigment epithelial detachments. *Lower panel* shows a cross-section optical coherence tomography image showing residual serous pigment epithelial detachments with complete resolution of the overlying subretinal fluid

Fluorescein angiography (Fig. 1c, d) revealed pooling of dye within each of the pigment epithelial detachments within the macula of both eyes. In the late phase of the angiogram, dye was found leaking into the subretinal space in her right eye. No edema or leakage from the discs was observed in either eye. Optical coherence tomography (Figs. 2 and 3) showed a large serous retinal detachment and multiple pigment epithelial detachments in her right eye and a pigment epithelial detachment without subretinal fluid in her left eye. The clinical diagnosis of idiopathic CSR was made and was attributed to our patient’s use of the oral fluocinonide gel. She was instructed to discontinue using the oral corticosteroid at the discretion of her dentist.

Two months following cessation of the oral topical corticosteroid gel, she reported an increase in vision in her right eye. Her best corrected visual acuity measured 20/25 in her right eye and 20/20- in her left eye. Posterior segment showed complete resolution of the subretinal fluid with residual pigment epithelial detachments in her right eye and stable pigment epithelial detachments in her left eye.

At her 6-month return visit, best corrected visual acuity measured at 20/20 in both eyes. An ocular examination showed stable pigment epithelial detachments and no evidence of recurrence of serous retinal detachments.

## Discussion

The use of glucocorticosteroids has been associated with the development of CSR [6–17]. Topical corticosteroid medications have been classified from I–VII depending on relative potency with I being the most potent and VII being the least [12]. We describe a patient who developed CSR while using oral topical fluocinonide gel 0.05%, a class II potent steroid. CSR has been shown to occur in patients using topical corticosteroids over a wide range of potency including high (our patient) and low potency class VII [15]. We offer a similar classification for oral topical corticosteroid applications (Table 1).

**Table 1:** Potency ranking of some commonly used topical dental corticosteroids

Class/Potency	Formulation	Brand-name
Class I: Highest potency		
Betamethasone dipropionate 0.05%	Ointment/Gel/Lotion	Diprogenta® 0.05% cream, Diprosone® 0.05% ointment/cream
Clobetasol propionate 0.05%	Ointment	Temovate® 0.05% ointment mixed in Orabase®
Fluocinolone acetonide 0.1%	Cream/Ointment	Compound mixed in Orabase®
Halobetasol propionate 0.05%	Cream/Ointment	Ultravate® 0.05% ointment mixed in Orabase®
Betamethasone sodium phosphate 0.5 mg per 5 ml	Oral rinse	Betamethasone sodium phosphate 0.5 mg mixed in 5 ml water
Class II: Higher potency		
Fluocinolone acetonide 0.05%	Gel/Ointment	Lidex® mixed in Orabase®
Mometasone furoate 0.1%	Cream	Elocom® 0.1% cream
Fluticasone propionate 0.05%	Cream	Cutivate® 0.05% cream
Fluticasone propionate 0.005%	Ointment	Cutivate® 0.005% ointment
Class III: Moderate potency		
Betamethasone valerate 0.05–0.1%	Cream/Ointment	Betnovate®, Diprosone®, Valisone® 0.1% ointment mixed in Orabase®
Triamcinolone acetonide 0.1%	Dental paste	Oracort® 0.1% paste, Kenalog® 0.1% mixed in Orabase®
Fluocinolone acetonide 0.025%	Ointment/Gel/Cream	Abricort® 0.025% ointment, Flucinar® 0.025% ointment, Synalar® 0.025% ointment
Alclometasone dipropionate 0.05%	Ointment/Cream	Aclovate® 0.05% ointment mixed in Orabase®
Class IV: Lower potency		
Fluocinolone acetonide 0.01%	Solution	Synalar® 0.01% ointment
Hydrocortisone 1.0–2.5%	Cream/Ointment, lotion	Hydrocortisone 1.0–2.5% cream
Dexamethasone 0.1%	Elixir/Solution	Rinsed or mixed in Orabase®

There is not currently a precisely known mechanism for onset of CSR as the pathogenesis of the disease remains unclear. It has been suggested that there is a disturbance in the blood–retina barrier at the posterior pole of the fundus within the choroidal vasculature, Bruch’s membrane, or retinal pigment epithelium (RPE). The introduction of indocyanine green angiography has added to our understanding of the disease, as it demonstrates the occurrence of diffuse hyperpermeability of the choroid associated with CSR [18]. Corticosteroids are known to inhibit collagen formation, a key component of Bruch’s membrane [19]. They may also cause dysfunction of the RPE by altering the transepithelial resistance to water and ions [20]. Disruption in the regulation of blood flow in the choroid is also possible as they may alter production of nitric oxide, prostaglandins, and free radicals [17, 20].

Our patient presented with subfoveal pigment epithelial detachments associated with her CSR. As with most cases of CSR associated with exogenous steroid use, conservative management for CSR with pigment epithelial detachments has been advocated since the majority of cases resolve with cessation of the offending medication. According to Mudvari *et al*, 22 of 34 patients (65%) with CSR and a pigment epithelial detachment who stopped using their corticosteroid had resolution of subretinal fluid and improved vision [21]. In this retrospective study, the length of time for resolution was variable from 6 months to 10 years and the mean visual acuity of these patients improved from 20/32 to 20/25. There have been several reports that focal laser, photodynamic therapy, eplerenone [22], spironolactone [23], topical NSAIDs [24], and intravitreal anti-vascular endothelial growth factor (VEGF) medications may have some treatment benefit [2, 25–28]. These treatments may offer a potential benefit for patients with chronic CSR or who are unable to discontinue their steroid medication.

To the best of our knowledge, one other published case of CSR accompanying the administration of oral topical corticosteroid exists in the literature, wherein George and Balan reported that a patient using 0.1% triamcinolone acetonide, a less potent medication, showed significant improvement in ocular symptoms following discontinuation of the oral gel 2 months later [16].

With regards to topical skin corticosteroids, there are multiple reported cases of topical steroids prescribed in dermatology associated with appearance of CSR [12–15]. Fernandez and colleagues reported a case of CSR in a patient using topical mometasone furoate cream 0.1% for lichen planus on her arms for 1 month [12]. Chan *et al.* demonstrated a temporal relationship with the presentation and recurrence of CSR [13]. They reported two cases of topical dermal corticosteroid use and CSR. The first case was a 64-year-old woman who was treated with betamethasone dipropionate 0.05% ointment for palmoplantar pustulosis, and the second case was a 56-year-old woman treated with a combination of clobetasol propionate 0.05% cream and betamethasone valerate 0.1% ointment for psoriatic plaque. In both cases, utilization of topical corticosteroid led to the development of signs and symptoms of CSR. Likewise, in both cases, CSR resolved upon discontinuation of the steroid. However, when patients were re-challenged with topical steroids, the CSR returned. Eventually, both cases were managed with orally administered tacrolimus 0.1% without any recurrence of CSR, as corticosteroids were no longer used [13].

The bioavailability of medications can vary by virtue of their route of administration. Medications that are taken via intravenous, intranasal, or sublingual routes may be more potent than those which are taken orally. When applied to oral mucosa or skin, the extent and rapidity of drug absorption can vary. For example, forearms absorb 1%, armpits 4%, face 7%, palms and soles < 0.1%, and mucosal regions such as eyelids and genitals 30% [12]. The oral mucosa is 4 to 4000 times more permeable than skin [29]. Damaged mucosal tissue has been associated with increased absorption and therefore increased incidence of adverse effects [29]. Such preparations of medications given through intranasal, sublingual, and mucosal routes may enter directly into the bloodstream through small capillaries residing in mucous membranes. Each of these parenteral routes bypass the initial metabolic breakdown that occurs in the liver known as the first pass effect and may potentially have a greater effect [30].

Plemons *et al.* conducted a study on the suppression of the adrenal system of patients with damaged oral mucosa by oral topical corticosteroid medication [31]. They proposed that patients with desquamative diseases of the gingiva and oral mucosa were at higher risk of adverse events related to increased systemic absorption of fluocinonide 0.05% gel, as the intact stratum corneum of the skin may act as a barrier against systemic absorption [31]. Although their findings did not show evidence of cortisol suppression or significant increases in systemic fluocinonide concentration, the authors postulated that it is possible that the concentrations in the systemic circulation were smaller than the detectable ranges or that fluocinonide was broken down into inactive metabolites [31]. In our patient, it is possible that the concentration of fluocinonide may have been strong enough to lead to CSR, but fortunately not strong enough to elicit cortisol suppression.

## Conclusion

To the best of our knowledge, this is the first case of idiopathic CSR associated with the use of topical oral fluocinonide gel. In theory, CSR may occur in patients taking any of the oral topical steroid medications commercially available. Discontinuing the use of oral corticosteroids may be associated with the resolution of CSR and improve vision in the affected patient. Doctors, including dentists and dermatologists, who commonly prescribe topical oral steroids should be informed of this possible association and should educate their patients about the potential side effects of these medications.

## Data Availability

The authors agree to make the images and data described in the manuscript freely available for use.
